# Quantitative Description of External Force Induced Phase Transformation in Silicon–Manganese (Si–Mn) Transformation Induced Plasticity (TRIP) Steels

**DOI:** 10.3390/ma12223781

**Published:** 2019-11-18

**Authors:** Zhongping He, Huachu Liu, Zhenyu Zhu, Weisen Zheng, Yanlin He, Lin Li

**Affiliations:** 1 School of Mechanical Engineering, Chengdu University, Chengdu 610106, China; sduhzp@hotmail.com; 2 School of Materials Science and Engineering, Shanghai University, Shanghai 200072, China; Liuhuachu@shu.edu.cn (H.L.); wszheng@shu.edu.cn (W.Z.); liling@i.shu.edu.cn (L.L.); 3 Advanced Research Institute, Chengdu University, Chengdu 610106, China; mse-zzy@live.com

**Keywords:** quantitative description, TRIP steel, retained austenite, phase transformation

## Abstract

Transformation Induced Plasticity (TRIP) steels with silicon–manganese (Si–Mn) as the main element have attracted a lot of attention and great interest from steel companies due to their low price, high strength, and high plasticity. Retained austenite is of primary importance as the source of high strength and high plasticity in Si–Mn TRIP steels. In this work, the cold rolled sheets of Si–Mn low carbon steel were treated with TRIP and Dual Phase (DP) treatment respectively. Then, the microstructure and composition of the Si–Mn low carbon steel were observed and tested. The static tensile test of TRIP steel and DP steel was carried out by a CMT5305 electronic universal testing machine. The self-built true stress–strain curve model of TRIP steel was verified. The simulation results were in good agreement with the experimental results. In addition, the phase transformation energy of retained austenite and the work borne by austenite in the sample during static stretching were calculated. The work done by austenite was 14.5 J, which was negligible compared with the total work of 217.8 J. The phase transformation energy absorption of retained austenite in the sample was 9.12 J. The role of retained austenite in TRIP steel is the absorption of excess energy at the key place where the fracture will occur, thereby increasing the elongation, so that the ferrite and bainite in the TRIP steel can absorb energy for a longer time and withstand more energy.

## 1. Introduction

As early as the 1940s, scholars paid attention to the influence of stress on phase transformation in steel. For example, in 1948, Guarnieri and Kanter [1] studied the internal stress of alloy steel in large castings to accelerate the bainite transformation of retained austenite. Howard and Cohen [2] reported in the same year that the formation of martensite promoted the transformation of austenite to bainite. In 1949, Jepson and Thompson [3] systematically revealed that the external stress of eutectoid steel (especially tensile stress) accelerates austenite isothermal decomposition. In 1953, Ko [4] found that the surface of steel sample is prone to bainite transformation indicating that the internal pressure is released and is conducive to the expansion phase transformation. In 1967, Zackay et al. [5] found the effect of stress-induced phase transformation promoted retained austenite to martensite in high alloy chromium–nickel–molybdenum steel. However, the high alloy chromium–nickel–molybdenum steel was too expensive with difficult deformation at medium temperature resulting in the steel not being widely used. In the past two decades [6,7,8,9,10,11], TRIP steels with silicon–manganese as the main element have attracted a lot of attention and great interest from steel companies due to the steel’s low price, high strength, and high plasticity.

A large number of scholars [12,13,14,15,16] have done a lot of research on TRIP steels involving the composition, heat treatment process, organization, grain size, and performance of TRIP steels. Retained austenite is the source of the high strength and high plasticity of TRIP steels, which is, of course, important to research. The principle of phase transformation plasticity has been widely recognized. The residual austenite which is stable at room temperature is transformed into martensite after being stressed, the transformed martensite increases the strength of the material, and the transient phase change induces plastic growth. This increases the strength and toughness of the steel. In addition, a large number of researchers [17,18,19,20,21,22] have studied the size, morphology, and quantity of retained austenite. However, the quantitative study of retained austenite in mathematics is minimal. 

Perlade et al. [23] divided TRIP steel into two parts: ferrite–bainite and martensite–austenite and then analyzed the relative proportion of each phase based on Olson–Cohen’s residual austenite transformation kinetics model. By applying the mixing rule of stress and strain and the law of equal work Perlade et al. obtained a constitutive model of the stress–strain relationship of TRIP steel. Bouquerel et al. [24] divided TRIP steel into two phases, ferrite and bainite, and then decomposed bainite into bainitic ferrite and martensite–austenite. A mixed structure of martensite and austenite was divided into retained austenite and strain-induced martensite. Finally, by successively applying the Gladman-type stress mixing rule, the above phases were combined to obtain a constitutive relation. All of them did not model the ferrite, bainite, martensite, and austenite phases, respectively. Choi et al. [25] assumed that ferrite, bainite, retained austenite, and martensite phases followed elastic–plastic isotropic hardening behavior under loading. Initial yield strengths, hardening coefficients, and hardening exponents of four phases were obtained. However, they did not consider the influence of chemical composition on relevant parameters and the energy absorption of retained austenite during phase transformation. The present work establishes the stress–strain models of ferrite, martensite, bainite, and austenite respectively, and then develops a stress–strain model for TRIP steel using the rule of mixtures. In this way, the energy absorption of residual austenite during phase transformation can be calculated. Finally, the energy of martensitic transformation after retained austenite is stressed can be mathematically analyzed.

## 2. Simulation of The TRIP Stress–Strain Curves

The relationship between true stress and true strain of polycrystalline materials can be expressed through Hollomon’s Equation [26]

(1) $$\sigma =K\varepsilon^{n}$$

where, *σ* is the true stress, *ε* is the true strain, *K* is the strength coefficient, and n is the strain hardening exponent. The Equation is based on the assumption that entire materials would be homogenized. However, the Equation is not entirely consistent with actual situations. If every phase of the structural materials can be expressed by Hollomon’s Equation, then the resulting simulation will be closer to a practical situation.

Kelly [27] pointed out that when strong continuous fires are embedded in a matrix which has a yield stress for plastic flow much less than the breaking stress of the fibers, the tensile strains in fiber and matrix are taken to be equal as follows

(2) $$\sigma =V_{f}\sigma_{f}+\left(1-V_{f}\right)\sigma_{M}$$

where *σ* is the true stress, $\sigma_{f}$ is the fiber true stress, $\sigma_{M}$ is the matrix true stress, and $V_{f}$ is the volume fraction of the fiber.

Davies [28] found that the mixture law of composites of two ductile phases is applicable to dual phase structures. The yield strength, the tensile strength, and the flow stress under different stress levels of DP steels conform to the mixture law that says that there is a linear relationship between the martensite volume fraction and the tensile strength or the flow stress. Yu [29] expanded this mixture law from DP steels to four-phase TRIP steels. The true stress–strain Equation of TRIP steels shows as follows.

(3) $$\sigma =V_{F}K_{F}\varepsilon^{n_{F}}+V_{M}K_{M}\varepsilon^{n_{M}}+V_{B}K_{B}\varepsilon^{n_{B}}+V_{Ar}K_{Ar}\varepsilon^{n_{Ar}}$$

where *σ* is the true stress; *ε* is the true strain; V_F_, V_M_, V_B_, and V_Ar_ are the volume fractions of ferrite, martensite, bainite, and austenite; K_F_, K_M_, K_B_, and K_Ar_ are the strength coefficients of ferrite, martensite, bainite, and austenite; and n_F_, n_M_, n_B_, n_Ar_ are the hardening exponents of ferrite, martensite, bainite, and austenite.

Research [30,31,32] shows that the carbon, silicon, and manganese contents affect K and n. This paper considered the effects of carbon, silicon, and manganese on ferrite, martensite, bainite, and austenite. Then,

(4) $$K_{i}=K_{0i}+A\left(C\%\right)+B\left(Mn\%\right)+C\left(Si\%\right)K$$

(5) $$n_{i}=n_{0i}+D\left(C\%\right)+E\left(Mn\%\right)+F\left(Si\%\right)n$$

where *i* represents ferrite–martensite–bainite–austenite and C%, Mn%, Si% are the chemical compositions of carbon, manganese, and silicon.

Based on the experiment results [33,34,35,36,37,38], the true stress–strain curve models of TRIP steels in a static state were gained. The model is

(6) $$\sigma =V_{F}\left(510+x\right)\varepsilon^{\left(0.23-y\right)}+V_{M}\left(2259+x\right)\varepsilon^{\left(0.26-y\right)}+V_{B}\left(659+x\right)\varepsilon^{\left(0.31-y\right)}\;+V_{Ar}\left(770+x\right)\varepsilon^{\left(0.36-y\right)}$$

(7) x=1643(C%)+63(Mn%)+109(Si%)

(8) y=0.079(C%)+0.024(Mn%)+0.037(Si%)

where *σ* is the true stress, *ε* is the true strain; V_F_, V_M_, V_B_, V_Ar_ represent the volume fractions of ferrite, matensite, bainite, and austenite; and C%, Mn%, Si% represent the chemical compositions of carbon, manganese and silicon.

**Figure 1:**
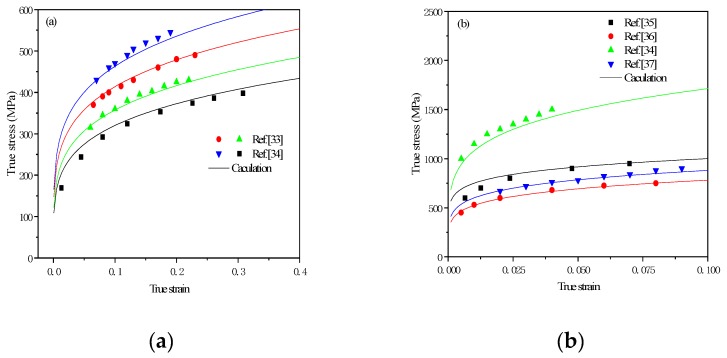

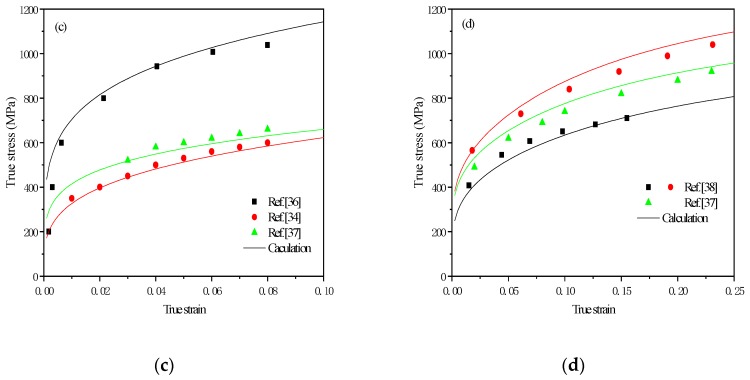
Calculation results and experiment results: (**a**) Ferrite; (**b**) Ferrite–Martensite; (**c**) Ferrite–Martensite–Bainite; (**d**) Ferrite–Martensite–Bainite–Austenite.

As seen in Figure 1, the results of the simulation and experiment are very close, which means that the model can simulate experimental results. As seen in Figure 1b, the [34] line is very high because there is only martensite in steel.

## 3. Experimental Procedure

The composition of cold rolled Si–Mn steel, provided by Baosteel, is shown in Table 1. For the convenience of calculation, trace elements such as P, S, Al, and N are not reflected in the calculations later. The thickness of TRIP and DP sheet steel is about 1.5mm.

**Figure 2:**
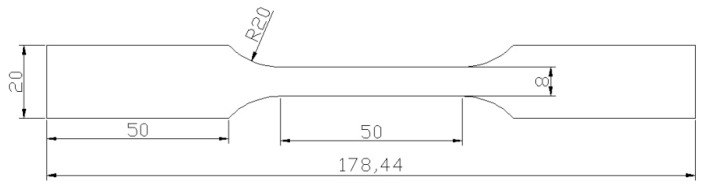
Quasi-static tensile specimens with thickness 1.5mm (unit=mm).

Specimens were machined in the rolling direction, as seen in Figure 2, and then heat-treated in salt bath furnaces. The TRIP steel material was processed following several steps: the first step was an intercritical annealing at 1058 K for 180 s, the second step was an isothermal bainitic transformation at 698 K for 180 s, then water quenching to room temperature [39,40]. The DP steel material was water quenched to room temperature after intercritical annealing at 1058 K for 180 s.

The microstructures of the two samples were observed by a metallographic microscope Nikon epiphot300 (Nikon, Tokyo, Japan) and a Hitachi SU-1510 tungsten filament scanning electron microscope (Hitachi, Tokyo, Japan). The phase composition was quantitatively analyzed by Image J analysis software (NIH, Bethesda, MD, USA) using grid method. The X-350A type X-ray diffraction (ST, Handan, China) measured the residual austenite content in the TRIP steel and the carbon content in the retained austenite. In this test, a Cr–Kα ray was used and the volume fraction of retained austenite was determined by the integrated intensity of the (220)γ and (211)α diffraction peaks. The Equation [41,42] for calculating the carbon content in retained austenite is

(9) $$C_{\gamma }=69.3383/sin\theta -75.9465C$$

where *θ* is the Bragg angle of the (220) crystal plane diffraction line.

The static tensile test was performed on a CMT5305 electronic universal testing machine (MTS, Eden Prairie, MN, USA) with a gauge length of 50 mm. The structure of the sample is shown in Figure 2.

The Ms temperature of the retained austenite in the TRIP steel was determined by the internal friction instrument (LMA, Suzhou, China). The internal friction test was carried out using a free vibration Ge’s inverted torsion pendulum instrument (LMA, Suzhou, China) with a sample size of 0.8 mm × 10 mm × 55 mm. The vibration frequency was 28 Hz and the cooling speed was 0.6 K/s.

## 4. Results and Discussion

### 4.1. Microstructure

**Figure 3:**
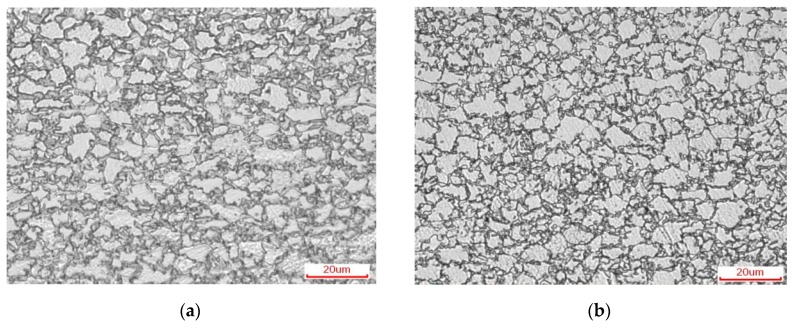

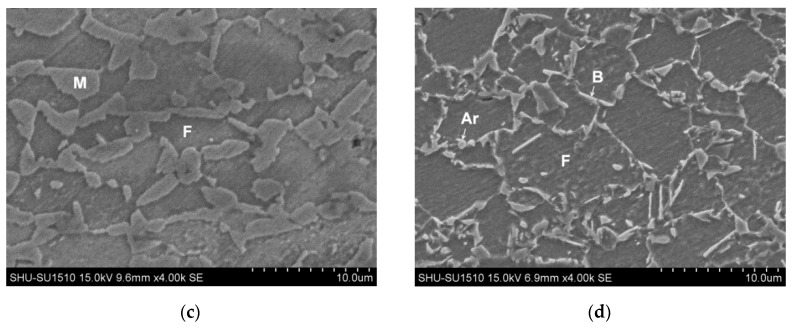
Optical micrographs of (**a**) Dual Purpose (DP) steel; (**b**) Transformation Induced Plasticity (TRIP) steel and SEM micrographs of (**c**) DP steel; (**d**) TRIP steel.

Figure 3 is an optical microstructure and SEM organization of experimentally obtained DP steel and TRIP steel. In Figure 3a, grayish black is martensite and white is ferrite. In Figure 3b, grayish black is bainite and retained austenite and white is ferrite. The content of each phase (volume fraction) of the two materials was obtained by quantitative metallographic analysis (grid method) and X-ray diffraction experiments. The micro-structure of DP steel consists of 60.8% ferrite and 39.2% martensite. The micro-structure of TRIP steel consists of 60.4% ferrite, 10.3% austenite, and 29.3% bainite.

After DP treatment the martensite in the steel distributed on the ferrite matrix in an island shape. As shown in Figure 3c, most of the martensite distributed on the ferrite grain boundary, with only a small amount of martensite particles distributed in the ferrite grain. In Figure 3d, the grayish black is the ferrite matrix, with bainite, and the retained austenite distributed on the ferrite matrix. Most of the retained austenite distributed at the ferrite grain boundary is in an island shape, with only a few retained austenite distributed in the ferrite grains, and bainite distributed at the ferrite grain boundary adjacent to the retained austenite.

**Figure 4:**
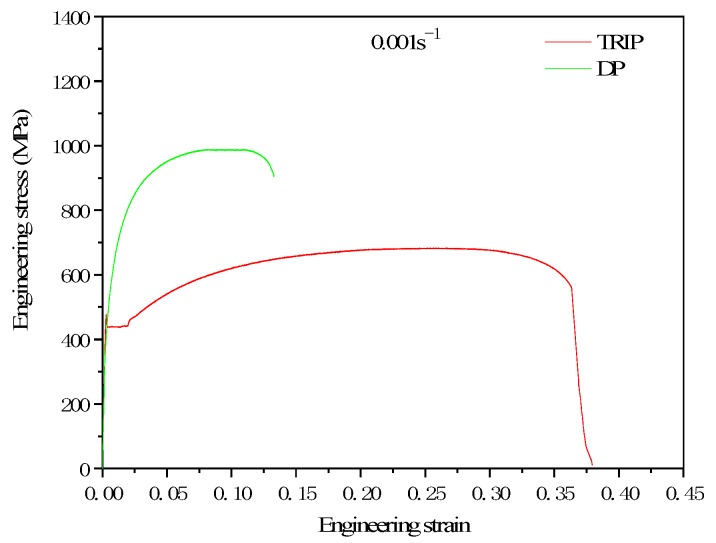
Tensile stress–strain curves of specimens after Dual Process (DP) and Transformation Induced Plasticity (TRIP) heat treatments.

Figure 4 shows the static tensile curves of TRIP steel and DP steel. It can be seen from the Figure that DP steel has continuous yielding characteristics, while TRIP steel has an obvious yield plateau caused by the formation of a Cottrell atmosphere as the effect of free carbon and dislocation. It considers that the carbon content in ferrite determines the yield platform’s appearance and disappearance. Shi [43] points out that as the carbon content increases, the yielding platform becomes shorter or even disappears. In this experiment, since the TRIP steel was kept at 1058 K for 3 min and then kept at 698 K for 3 min, secondary carburization occurred while DP steel was only critically annealed at 1058 K for 3 min. The process resulted in more ferritic–carbon content in DP steel than TRIP steel, so TRIP steel produced a yielding platform, while DP steel did not.

**Figure 5:**
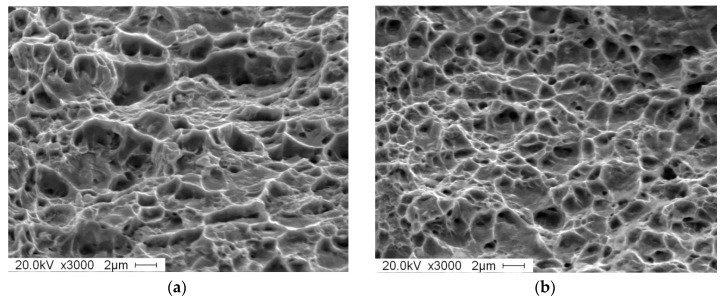
Surface of specimens after two heat treatments: (**a**)Dual Process (DP) steel; (**b**) Transformation Induced Plasticity (TRIP) steel.

As seen in Figure 4, the elongation of TRIP steel is almost three times than of DP steel. The reason can be seen in Figure 5, where the fracture morphology of TRIP steel is deeper than DP steel and the fracture morphology in TRIP steel is much greater than in DP steel. TRIP steel reflects very good toughness fracture characteristics.

### 4.2. Calculation

As seen in Table 2, the chemical composition of ferrite and austenite in the cold-roll steel sheets after holding at 1058 K for a period of time was calculated by Software Thermo-Calc using the TCFE9 database. DP steel was water quenched to room temperature after intercritical annealing at 1058 K for 180 s. Table 2 also includes the chemical composition of DP steel.

After holding the materials at 698 K for a period of time, the chemical composition of TRIP steels are shown in Table 3. The volume fraction of ferrite is constant. The volume fraction of austenite is 10% as measured by X-ray diffraction(XRD). The rest is 30% bainite. It is supposed that the contents of both Si and Mn are constant in the retained austenite and bainite. The content of C in the retained austenite is 0.95% as calculated by Equation 9 measured by XRD. The rest of the carbon is in the bainite. After tensile progress, phase transformation happened and the martensite appeared. Martensite comes from austenite by shear deformation, so they have the same chemical composition.

The relation between retained austenite and true strain was expressed in author’s article [44] and the model shows as follows

(10) $$V=\left(1.431+0.825V_{0}\right)e^{\left(-7.56-0.69W_{C}\right)\varepsilon }$$

where *V* is the volume of retained austenite, V_0_ is the volume of initial retained austenite, *e* is the hardening exponents, *W_c_* is the chemical composition of carbon in retained austenite, and *ε* is the true strain.

By putting the chemical composition of each phase in Table 2, Table 3, and Equation (10) into Equation (6), the true stress–strain curve models of DP steel and TRIP steel are obtained.

DP steel

(11) $$\sigma =0.60*706\varepsilon^{0.16}+0.40*2957\varepsilon^{0.14}$$

TRIP steel

(12) $$\sigma =0.60*706\varepsilon^{0.17}+0.30*1010\varepsilon^{0.21}+V_{Ar}*1120\varepsilon^{0.19}+V_{M}*4088\varepsilon^{0.09}$$

(13) $$V_{Ar}=\left(1.431+0.825*10\right)e^{\left(-7.56-0.69*0.95\right)\varepsilon }$$

(14) $$V_{M}=0.10-\left(1.431+0.825*10\right)e^{\left(-7.56-0.69*0.95\right)\varepsilon }$$

where *σ* is the true stress and *ε* is the true strain.

**Figure 6:**
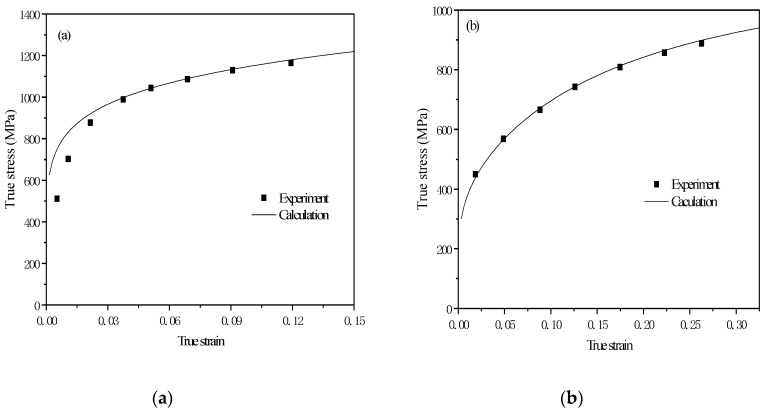
Calculation result and experiment result: (**a**)Dual Process (DP) steel; (**b**)Transformation Induced Plasticity (TRIP) steel.

Figure 6 is the comparison between the experiment results in the true stress–strain curves of DP steel and TRIP steel which is formed through different heat treatment of Si–Mn low carbon steel cold-rolled sheet and the result of calculation from the true stress–strain curves model of DP steel and TRIP steel. Based on Figure 6, the results of the simulation and experiment are very close, which means that the model can simulate the experimental results. From Equation (12), it can be seen that in TRIP steel, martensite has the highest strength. The strength of bainite and austenite are closed. Ferrite has the lowest strength.

**Figure 7:**
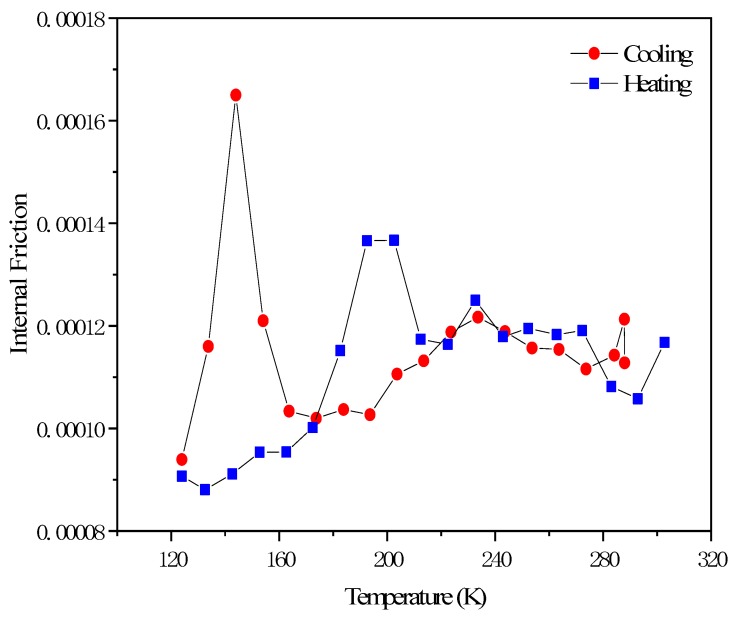
TRIP steel: Internal friction-temperature spectrum.

Figure 7 shows the Ms temperature measured by the internal friction instrument. The measurement process is first to cool down (red line) and then increase (blue line) the temperature. The temperature measured by the internal friction instrument is 144 K with a cooling rate of 0.6 K/min. 

According to the author’s article [45], the calculation model of Ms temperature in Fe–C–Mn–Si alloy is

(15) $$\begin{array}{ll} \Delta G_{Fe-C-Mn-Si}^{\gamma \to M}= & \Delta G_{Fe-C-Mn-Si}^{\gamma \to \alpha }+1200+8500\chi_{C}+8000\chi_{Mn}+6000\chi_{Si}+20000\chi_{C}\chi_{Mn}\\ & +18000\chi_{C}\chi_{Si}+16000\chi_{Mn}\chi_{Si}+7\times 10^{7}\chi_{C}\chi_{Mn}\chi_{Si}=0 \end{array}$$

where $\Delta G_{Fe-C-Mn-Si}^{\gamma \to M}$ is martensitic critical phase transformation driving force of Fe–C–Mn–Si alloy, $\Delta G_{Fe-C-Mn-Si}^{\gamma \to \alpha }$ is Gibbs free energy for austenite to ferrite transformation of Fe–C–Mn–Si alloy in equilibrium, and $\chi_{C}$, $\chi_{Mn}$ and $\chi_{Si}$ are the mole fraction of carbon, manganese, and silicon.

Through the Thermo-Calc software TCFE9 database and TQ interface, the Ms temperature of the retained austenite in the TRIP steel of this experiment was finally calculated to be 143.8 K. Comparing the experimental results with the calculated results of the two being very close, indicating that the calculated model is feasible. In order to cause the martensite transformation of the retained austenite at room temperature of 298 K, $\Delta G_{Fe-C-Mn-Si}^{\gamma \to M}$ is calculated as 1000 J/mol.

The average molar mass of TRIP steel can be calculated by the Equation as follows

(16) $$M=1/(W_{Fe}/M_{Fe}+W_{C}/M_{C}+W_{Mn}/M_{Mn}+W_{Si}/M_{Si})$$

where *M* is the average molar mass and *W_Fe_*, *W_C_*, *W_Mn_* and *W_Si_* are the mass fractions of iron, carbon, manganese, and silicon. The average molar mass of the cold rolled steel was calculated as 56.28 g/mol.

The amount of substance by external force in the tensile specimen can be calculated by the equation as follows

(17) n=ρ∗V/56.28n=ρ∗V/56.28

where n is the amount of substance by external force, ρρ is the density of TRIP steel, and V is the volume of substance by external force. The amount of substance by external force was calculated as 0.138 mol.

As known from the microstructure of TRIP steel, the volume fraction of retained austenite is 10.3% and the amount of retained austenite is 0.0142 mol. The transformation percentage of residual austenite was calculated as 67.7% by Equation (10). The amount of retained austenite transformation was 0.00912 mol. Therefore, the total retained austenite of all transitions in the TRIP steel required work of 9.12 J.

The work by tensile force based on the experimental stress–strain curve is calculated with the following Equation

(18) $$W=V\int^{\text{​}}\sigma d\varepsilon W=V\int \sigma d\varepsilon$$

where *W* is the work by tensile force, *V* is the volume of deformed part, *σ* is the true stress, and *ε* is the true strain. The work by tensile force was calculated as 215.8 J. The total work is 217.8 J which was calculated by the TRIP steel true stress–strain curve model in Equation (12). Comparing the experimental results with the calculated results the two are very close, indicating that the true stress–strain curve model of TRIP steel is very accurate.

Through the models of various microstructures, such as ferrite, bainite, austenite, and martensite, it was calculated that the work borne by ferrite is 68.6 J and the work borne by bainite is 98 J. The work borne by austenite–martensite is 51.2 J, in which the work borne by martensite is 36.7 J and the work borne by austenite is 14.5 J. As known from the above, the phase transformation energy absorption of retained austenite in the sample is 9.12 J. So, the real work done by austenite is only 5.38 J.

The work done by DP steel in the stress–strain curve experimental data is 99.1 J. The total work is 95.6 J as calculated by the true stress–strain curve model of DP steel in Equation (11). Comparing the experimental results with the calculation results, the two are very close. It shows that the true stress–strain curve model is very accurate. Through the model of the ferrite and martensite phase, it was calculated that the work of ferrite is 33.8 J and the work of martensite is 61.8 J.

Comparing the work done by microstructures in TRIP steel and DP steel shows that the same volume fraction of ferrite has a total work capacity of 68.6 J in TRIP steel and only 33.8 J in DP steel. The work in TRIP steel is twice that of DP steel, which is related to the different elongations between the two steels. Premature fracture of DP steel greatly reduces the work done by ferrite in the steel.

The work done by austenite is very small, only 14.5 J, accounting for 6.66% of the total energy. The phase transformation energy absorption of retained austenite in the sample is 9.12 J, only accounting for 4.19% of the total energy. The energy in TRIP steel is mainly borne by ferrite and bainite. The energy consumed by these two materials accounts for 76.49% of the total energy. The role of retained austenite is not the absorption of total energy but the absorption of energy at key parts. When stress concentration occurs in a certain part, the retained austenite will undergo a phase change, absorb excess energy, delay the time of breaking, and ultimately increase elongation.

## 5. Conclusions

In this study, the cold rolled sheets of Si–Mn low carbon TRIP and DP steel were studied. The true stress–strain curve models of TRIP and DP steel were established. The phase transformation energy of retained austenite and the work borne by austenite in TRIP steel during static stretching were calculated. The main conclusions are as follows:

(1) The static true stress–strain curve model of TRIP steel was established. The curve calculated by this model is in good agreement with experimental data.

(2) It can be found that in TRIP steel, martensite has the highest strength. The strength of bainite and retained austenite are close. Ferrite has the lowest strength.

(3) The work done by austenite is 14.5 J, which is negligible compared to the total work of 217.8 J. The phase transformation energy absorption of retained austenite in TRIP steel is 9.12 J. The effect of retained austenite is absorption of the energy at break. The increased elongation allows the ferrite and bainite in TRIP steel to absorb energy for a longer period of time and to withstand more energy.

## Figures and Tables

**Table 1:** Chemical composition of specimens (wt%).

Steel	C	Si	Mn	P	S	Al	N	Heat Treatment Process
Transformation of Induced Plasticity (TRIP)	0.11	1.2	1.5	0.01	0.005	≤0.03	≤0.0035	1058 K × 3 min + 698 K × 3 min
Dual Process (DP)	0.11	1.2	1.5	0.01	0.005	≤0.03	≤0.0035	1058 K × 3 min

**Table 2:** Composition and volume fraction of specimens at 1058 K (wt%).

	C	Si	Mn	Vol (wt%)
Ferrite	0.0075	1.25	0.95	60
Austenite (Martensite)	0.262	1.12	2.32	40

**Table 3:** Composition and volume fraction of Transformation Induced Plasticity (TRIP) steel (wt%).

	C	Si	Mn	Vol (wt%)
Ferrite	0.0075	1.25	0.95	60
Bainite	0.0525	1.12	2.32	30
Retained austenite	0.95	1.12	2.32	10
Martensite	0.95	1.12	2.32	–
